# In vitro and in vivo BNCT investigations using a carborane containing sulfonamide targeting CAIX epitopes on malignant pleural mesothelioma and breast cancer cells

**DOI:** 10.1038/s41598-020-76370-1

**Published:** 2020-11-06

**Authors:** Diego Alberti, Alessia Michelotti, Alberto Lanfranco, Nicoletta Protti, Saverio Altieri, Annamaria Deagostino, Simonetta Geninatti Crich

**Affiliations:** 1 Department of Molecular Biotechnology and Health Sciences, University of Torino, Via Nizza 52, 10126 Turin, Italy; 2 Department of Chemistry, University of Torino, Via P. Giuria 7, 10125 Turin, Italy; 3 Department of Physics, University of Pavia, Via Agostino Bassi 6, 27100 Pavia, Italy; 4 Nuclear Physics National Institute (INFN), Unit of Pavia, Via Agostino Bassi 6, 27100 Pavia, Italy

**Keywords:** Cancer, Cancer therapy, Targeted therapies, Medicinal chemistry, Molecular medicine

## Abstract

This study aims at merging the therapeutic effects associated to the inhibition of Carbonic Anhydrase IX (CAIX), an essential enzyme overexpressed by cancer cells including mesothelioma and breast cancer, with those ones brought by the application of Boron Neutron Capture Therapy (BNCT). This task was pursued by designing a sulfonamido-functionalised-carborane (CA-SF) that acts simultaneously as CAIX inhibitor and boron delivery agent. The CAIX expression, measured by Western blot analysis, resulted high in both mesothelioma and breast tumours. This finding was exploited for the delivery of a therapeutic dose of boron (> 20 μg/g) to the cancer cells. The synergic cytotoxic effects operated by the enzymatic inhibition and neutron irradiation was evaluated in vitro on ZL34, AB22 and MCF7 cancer cells. Next, an in vivo model was prepared by subcutaneous injection of AB22 cells in Balb/c mice and CA-SF was administered as inclusion complex with a β-cyclodextrin oligomer. After irradiation with thermal neutrons tumour growth was evaluated for 25 days by MRI. The obtained results appear very promising as the tumour growth was definitively markedly lower in comparison to controls and the CAIX inhibitor alone. This approach appears promising and it call consideration for the design of new therapeutic routes to cure patients affected by this disease.

## Introduction

In the last decade, combinations of different therapeutic modalities have been under intense scrutiny as possible strategies to cure cancer^1^. This choice has been prompted by the observation that, although many chemotherapeutic protocols and radiotherapy treatments could yield to a significant decrease of tumour masses, invariantly they were unable to avoid tumour re-growth^2^. Moreover, the frequent occurrence of chemoresistance and radioresistance shown by a minor fraction of tumour cell and the nonspecific toxicity vs. normal cells are other major limitations of standard therapies^2–4^. Nowadays there is a growing consensus that (i) complete tumour regression may be achieved by combining different therapeutic strategies, and (ii) a better control of off-target toxicity can be tackled by developing target-specific drugs as well as by using a more specific radiotherapeutic protocol. An important class of targeted therapies addresses the use of substances that inhibit enzymes essential for cancer cells to grow. Among them, Carbonic Anhydrase IX (CAIX) has been under considerable attention. It is a hypoxia-inducible enzyme with extracellular catalytic domain that converts water and carbon dioxide into bicarbonate ions and protons. Overexpression of this enzyme in a variety of cancers including mesothelioma^4–7^ and breast cancer^8–10^ was reported. CAIX plays an important role in tumour acid–base homeostasis by promoting cancer cell survival also in hypoxic microenvironment. The role of CAIX consists in producing and maintaining an intracellular pH favorable to tumour cell growth and survival, while at the same time contributing to the generation of an increasingly acidic extracellular space that facilitates tumour cell invasiveness^11,12^. For these reasons, novel antitumour therapies based on the use of CAIX inhibitors (e.g. acetazolamide) are under intense scrutiny in clinic^9^. In this context, it has been already reported the use of sulfonamide functionalised carboranes, as CAIX inhibitors^13–16^. The introduction of the carborane structure (icosahedral lipophilic cluster containing Boron atoms) in the drug design is deemed to enhance the hydrophobic interactions of biologically active compounds with their receptors and to increase their in vivo stability and bioavailability^17–19^. In fact, o-carborane is an icosahedral cluster that possess 3D aromatic character^20,21^. This property is very important because, according with docking analysis, increases the affinity for the enzyme binding region. Moreover, it shows low catabolism and enzymatic degradation. As examples, 1,7-closo-carboranylanilinoquinazoline hybrids^22^ have been identified as EGFR inhibitors, some of them with higher affinity than the parent compound erlotinib and fluorinated carborane-containing estrogen receptor beta (ERβ) modulators showed an enhanced selectivity for ERβ^23^. In the specific case of CA inhibition, the use of functionalised carboranes increases the selectivity for CAIX overexpressing tumour cells thus reducing unwanted side effects. Furthermore, carboranes are used as agents in Boron Neutron Capture Therapy (BNCT), an example of targeted therapy with good efficacy and low toxicity that provides a tumour-selective cell death^24–26^. BNCT is a non-invasive therapeutic modality for treating locally invasive malignant tumours, successfully applied to primary brain tumours and recurrent head and neck cancer^27^. More specifically, this therapy combines low energy neutron irradiation with the presence of boron-containing compounds at the target cells. Neutrons are captured by nonradioactive ^10^B yielding ^11^B that disintegrates into alpha particles and lithium nuclei causing non reparable damage to the cell where they were generated, sparing the surrounding healthy ones. This fact makes BNCT a powerful option for the treatment of disseminated metastasis and infiltrating tumours as mesothelioma and invasive breast ductal carcinoma, whose cure cannot be tackled by methods, such as surgery or conventional radiotherapy, because of a non-sufficiently accurate localization of the tumour lesion. Another advantage of BNCT is related to the use of a PRE-targeting strategy by injecting a non-radioactive, non-toxic compound that will be activated only after its accumulation at the pathological site is considered sufficient for a successful therapy. Most of BNCT applications have dealt with skin melanoma, brain, and head and neck tumours^28^. Recently, in Japan some mesothelioma patients obtained significant improvement of the symptoms when undergone to BNCT^29,30^. *L*-para-boronphenylalanine (BPA), a mimic of the amino acid phenylalanine and sodium mercaptoundecaidro-*closo*-dodecaborate (BSH) are the only two boron containing compounds currently used in clinical applications. They permit to reach a boron concentration ratio between tumour and normal tissues in the range of 3–5. Nevertheless, it is known in the BNCT research community that an improvement of the selective uptake of boron containing agents into the targeting tumour cells is necessary to increase the number of clinical use of BNCT^31–34^. In this contesxt, our group recently developed a macromolecular dual MRI/BNCT agent able to deliver to mesothelioma cells a therapeutic dose of boron (26 μg/g), significantly higher than in the surrounding tissue (3.5 μg/g) by exploiting overexpressed Low Density Lipoproteins (LDL) receptors^35^. Furthermore, Nakamura and coworkers recently reported interesting results using a Hyaluronic acid containing BSH agent for the delivery of the boron payload to mesothelioma in mouse models^36^. BNCT was also proposed for the treatment of locoregional recurrences of HER2 + breast cancer subtype using immunoliposomes or LDL as boron carriers^37,38^.

**Figure 1:**
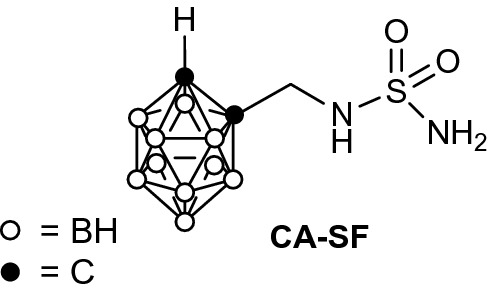
Structure of the carborane functionalised sulfonamide (CA-SF) used in this work.

The aim of our study was to explore a synergic therapeutic approach, arising from the combination of a preliminary enzymatic inhibition of CAIX by the carborane functionalised sulfamide (Fig. 1) followed by neutron irradiation. The design of the inhibitor relied on the consideration that almost all the most potent inhibitors of CAs contain a terminal sulfonamide able to coordinate to Zn^2+^ ions in the catalytic site of CAIX. Thus a single agent operates two roles both aimed at contrasting the tumour cell growth. This therapeutic approach can potentially affect only tumour cells with a lethal dose of radiations, even in the presence of spreading and infiltrative masses common in mesothelioma patients and in ductal adenocarcinoma. At present, the conventional radiotherapy cannot do such selective treatment because unavoidably significant masses of healthy tissue are exposed to high doses of radiation.

## Materials and methods

Flasks and all equipments used for the generation and reaction of moisture-sensitive compounds were dried by electric heater under Nitrogen. All commercially solvents and reagents were used as received. Decaborane was purchased from KATCHEM spol. s.r.o. Product were purified by preparative column chromatography on silica gel for flash chromatography, 0.04–0.063 mm/230–400 mesh.

Reactions were monitored by TLC using silica gel on TLC-PET foils Sigma-Aldrich, 2–25 mm, layer thickness 0.2 mm, medium pore diameter 60 Å. Carboranes and their derivatives were visualized on TLC plates using a 5% PdCl_2_ aqueous solution in HCl. ^1^H NMR spectra were recorded at 200 and 600 MHz, ^13^C NMR spectra at 50.2 and 150 MHz, in CDCl_3_ or (CD_3_)_2_CO. Data were reported as follows: chemical shifts in ppm from tetramethylsilane as internal standard, integration, multiplicity (s = singlet, d = doublet, t = triplet, q = quartet, dd = double-doublet, m = multiplet, br = broad), coupling constants (Hz), and assignment. ^13^C NMR spectra were measured with complete proton decoupling. ESI MS spectra were registered on a Waters micromass ZQ spectrometer equipped with ESI ion source. IR spectra were recorded on a Perkin Elmer BX FT-IR. Thiazolyl Blue Tetrazolium Bromide (MTT), Acetazolamide (AZ) (5-acetamido-1,3,4-thiadiazole-2-sulfonamide) were purchased by Sigma Aldrich. Poly-β-cyclodextrin were purchased by Cyclolab (Hungary).

### Synthesis of 1-Sulfonamidomethyl-***o***-carborane (CA-SF) and 1-sulfonamidomethyl-^10^B enriched-***o***-carborane (^10^B CA-SF)

**Scheme 1:**
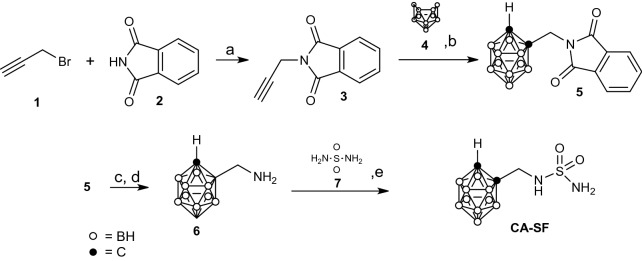
Synthesis of 1-sulfonamidomethyl-*o*-carborane (**CA-SF**) and 1-sulfonamidomethyl-^10^B enriched-*o*-carborane (^**10**^**BCA-SF**). *Reaction conditions and yields*: (**a**) K_2_CO_3_, acetone, reflux; (**b**) decaborane (0.7 eq), (bmim^+^) Cl^-^ (0.4 eq), anhydr. toluene, under Ar, 90 °C ; (**c**) NaBH_4_, *i*PrOH/H_2_O 4/1, under Ar, rt; (**d**) CH_3_COOH/HCl 4/1, rt; (**e**) Sulfonamide (5 eq), anhydr. dioxane, reflux.

The sulfamido carborane **CA-SF**, both natural boron abundant and ^10^B enriched, has been prepared by dehydrogenative insertion of *N*-phtalimidopropyne **3** and decaborane **4** followed by deprotection and reaction of the resulting carboranylamine **6** with sulfonamide **7**, Scheme 1. All spectroscopic data were consistent with those reported in literature^14,39,40^.

#### Propargyl phtalimide (3)^41^

Phtalimide (20.0 mmol, 2.94 g) was dissolved in 50 mL of acetone in a 100 mL round bottom flask, then K_2_CO_3_ (2.5 equiv, 50.0 mmol, 7.0 g) followed by propargyl bromide (40.0 mmol, 3.8 mL) were added. The reaction mixture was stirred at reflux overnight then quenched with a solution of 5% HCl and extracted with Et_2_O (3 × 30 mL). The combined extracts were washed with HCl 5% (1 × 30 mL) and brine, dried and evaporated under reduced pressure affording 0.80 g (21%) of a pale yellow oil which was at once used for the following reaction. δ_H_ (200 MHz; CDCl_3_, Me_4_Si): 2.23 (1H, t, *J* = 2.6 Hz, C*H*), 4.45 (2H, d, *J* = 2.6 Hz, C*H*_2_N), 7.80 (4H, m, Ar).

#### ***C-***phtalimidomethyl-***C′***-H-^10^B- enriched-***o***-carborane (5)

In a dried heavy wall tube containing a stirring bar, propargyl phtalimide **3** (3.5 mmol, 0.64 g) and ^10^B-enriched decaborane (2.3 mmol, 0.26 g) were reacted under Ar in a biphasic mixture of 0.25 g of (bmim)^+^Cl^−^ (1.4 mmol) and 5 mL of anhydrous toluene. The crude purification by column chromatograpy (CH_2_Cl_2_) afforded 0.29 g of **5** (45%). ν_max_ (neat)/cm^−1^ 2578, 1774, 1718, 1420, 1402, 1366, 719; δ_H_ (200 MHz; CDCl_3_, Me_4_Si): 0.40–4.40 (10H, m, B*H*), 4.00 (1H, br s, B_10_H_10_C*H*), 4.46 (2H, s, C*H*_2_N), 7.80 (4H, m, Ar); δ_c_ (50.2 MHz; CDCl_3_, Me_4_Si): 42.3 (CH_2_), 59.8 (CH), 72.9 (Cq), 123.9 (CH), 131.0 (Cq), 134.7 (CH), 167.0 (Cq).

#### 1-Methylamino-10B-enriched-***o***-carborane (6)

^41^*C-*phtalimidomethyl-*C*′-H-^10^B-enriched-*o*-carborane **(5)** (1.0 mmol, 0.27 g) was suspended in 8 mL of *i*PrOH in a 50 mL round bottom flask and NaBH_4_ was added (0.19 g, 5.0 mmol) and stirred overnight under Ar at room temperature. The solvent was evaporated and the obtained residue was dissolved in H_2_O, extracted with CH_2_Cl_2_ (1 × 10 mL) and Et_2_O (2 × 10 mL). The combined organic phases were dried with Na_2_SO_4_, filtered and concentrated. The resulting solid was dissolved in 10 mL of a mixture of CH_3_COOH/HCl (4/1) and the solution was stirred at room temperature for 2 h 30′. The solvent was evaporated and the residue was suspended in CHCl_3_ and stirred for 3 h at rt. Finally, the suspension was filtered and the recovered white solid was washed with CHCl_3_ and dried affording 0.17 g of **6** (99%). ν_max_ (neat)/cm^−1^ 2825, 2611, 2591, 1512, 738; δ_H_ (200 MHz; CDCl_3_, Me_4_Si): 1.00–3.50 (10H, m, B*H*), 4.69 (2H, s, C*H*_2_NH_2_), 5.97 (1H, br s, B_10_H_10_C*H*).

#### 1-Sulfonamidomethyl-^10^B-enriched-***o***-carborane (^10^B-enriched CA-SF)

In a 50 mL round bottom flask, 0.18 g of 1-methylamino-^10^B-enriched-*o*-carborane(**6**) (1.1 mmol, 0.18 g) and 5 eq of sulfonamide were suspended in 5 mL of anhydrous 1,4-dioxane and stirred at reflux for 2 h. The solvent was evaporated and the obtained residue was extracted with a mixture of Et_2_O/EtOAc 1/1 (3 × 10 mL), washed with a saturated solution of KHSO_4_ (1 × 10 mL) and brine (1 × 10 mL). The combined organic phases were dried with Na_2_SO_4_, filtered and concentrated. The crude was purified by column chromatography (CH_2_Cl_2_/CH_3_OH 95/5) affording 0.12 g of ^10^B-enriched CA-SF (0.49 mmol, 45%) as a white solid, m.p. 117–118 °C. ν_max_ (neat)/cm^−1^3377, 3275, 3054, 2581, 1590, 1543, 1455, 1432, 1347, 1155; δ_H_ (600 MHz; (CD_3_)CO, Me_4_Si): 1.72–2.57 (10H, m, B*H*), 3.87 (2H, d, *J* = 7.8 Hz, C*H*_2_NH), 4.58 (1H, br s, B_10_H_10_C*H*), 6.23 (2H, br s, N*H*_2_), 6.76 (1H, br t, *J* = 7.8 Hz, CH_2_N*H*). δ_c_ (150.2 MHz; CDCl_3_, Me_4_Si): 47.7 (CH_2_), 59.2 (CH), 75.2 (Cq), ESI HRMS for C_3_H_16_B_10_N_2_O_2_S Calcd: 243.2207 [M–H]^-^, Found: 243.2308 [M–H]^−^ All spectra are reported in Supporting Information.

#### Cell lines

MCF7 breast cancer cell line was obtained from the ATCC. The cells were cultured using the same protocol reported by Azzi et al^42^. Briefly, cells were seeded in flasks using EMEM medium (Lonza) supplemented with 10% (v/v) fetal bovine serum (FBS) (Lonza), 1 mM sodium pyruvate, 2 mM glutamine, non-essential amin o acids, 0.01 mg/mL human insulin (Sigma Aldrich). Mouse mesothelioma (AB22) cell line was obtained from Sigma Aldrich and they were cultured in RPMI medium (Lonza) supplemented with 25 mM Hepes, 10% (v/v) FBS and 2 mM glutamine. Human mesothelioma (ZL34) cell line was obtained from Sigma Aldrich. The cells were cultured in DMEM-Ham’s F12 (Lonza) containing 2.5 mM glutamine and supplemented with 15% (v/v) FBS using the same protocol described in reference^35^. All media contained 100 U/mL penicillin and 100 U/ml streptomycin. All the cell lines were maintained in a humidified incubator at 37 °C, 5% CO_2_.

### Western blot analysis

Western blots were carried out to show the expression of CAIX in MCF7, ZL34 and AB22 cultured in complete medium in condition of normoxia in a cell incubator at 37 °C, 5% CO_2_. For the Western blot analysis, 6 × 10^5^ MCF7, 1.5 × 10^6^ ZL34 and 2 × 10^5^ AE17 were seeded in 6 cm of diameter dishes. After 24 h for MCF7 and ZL34 cells and after 72 h for AB22, the cells were washed 3 times with PBS and then were detached using a cell scraper and frozen at − 80 °C until further analysis. Cells were re-suspended in 200uL of Ripa Lysis Buffer (Sigma-Aldrich) on ice in the presence of protease inhibitor cocktail (Sigma-Aldrich) and incubated for 30′ on ice. Cell lysates were centrifuged at 14,000×*g* for 5′ at 4 °C and the supernatants were collected and assayed for protein concentration with the BCA protein assay kit (Thermo Fisher Scientific). Cell lysates (60 µg of proteins) were denaturated at 96 °C for 3′ in the presence of 2 × Laemmli buffer (BioRad, Hercules, CA, USA) in a termomixer and then were separated on 4–15% gradient Mini-Protean precast gel (Biorad) and transferred to nitrocellulose membranes (BioRad) activated before use with methanol for 15 s. The blots were blocked with 5% nonfat dry milk in TTBS buffer (Tris–HCl 20 mM, Tween 20 0.05%, NaCl 500 mM, pH 7.5) for 2 h at RT and then incubated overnight at 4 °C with a 1:1300 dilution in 5% nonfat dry milk in TTBS buffer of polyclonal CA IX antibody (NB100-417; Novus, Littleton, Colo), washed 3 times in TTBS buffer and then incubated for 1 h at RT with a 1:2000 dilution of HRP-conjugated goat anti-rabbit Ig Ab (Sigma-Aldrich). Ab binding was visualised by Pierce ECL Western Blotting Substrate (Thermo Fisher Scientific) according to the manufacturer's instructions. Membranes were subsequently probed with an anti-actin Ab (Sigma-Aldrich) used as control for equal protein loading. Images were acquired with an ChemiDoc Touch Imaging System and analyzed with ImageJ software. Relative CAIX expression was calculated from the ratio between CAIX and actin density bands of each sample.

### MTT assay

The MTT assay is based on the reduction in the mitochondria of metabolically active cells by succinate dehydrogenase obtaining a precipitation of purple formazan crystal. The assay was carried out using the same protocol reported by Azzi et al^42^. Briefly, AB22, ZL34 and MCF7 cells were seeded at a density of 7 × 10^3^, 5 × 10^3^ and 1 × 10^4^ cells per well, respectively, in a 96-well microtiter plate. After 24 h at 37 °C and 5% CO_2_, they were incubated with increasing concentration of CA-SF or AZ for 24 h in a condition of normoxia at 37 °C and 5% CO_2_ by adding stock solutions in dimethyl sulfoxide (DMSO). In all the conditions the concentration of DMSO during the cell incubation was maintained under 0.25% (v/v) in cell medium. After the incubation, the medium was removed and each well was incubated with thiazolyl blue tetrazolium bromide (Sigma) dissolved in the medium at a concentration of 0.45 mg/mL for 4 h at 37 °C and 5% CO_2_. Then, after medium elimination, 150 µL of DMSO were added into each well to solubilise the formazan salt crystals produced by the metabolism of live cells and the microplate was incubated at room temperature (RT) for 30 min. Finally, absorbance was measured at 570 nm using an iMark microplate reader (Biorad). Cell vitality was reported as the percentage of dead cells observed in the treated samples relative to that observed in the non-treated control cells.

### Uptake experiments

For the in vitro uptake experiments, 6 × 10^5^ MCF7, 4.5 × 10^5^ ZL34 and 2 × 10^5^ AB22 were seeded in 6 cm of diameter dishes. After 24 h for MCF7 and ZL34 and after 72 h for AB22, the cells were incubated for 24 h with increasing concentration of CA-SF. All the incubations were performed in condition of normoxia at 37 °C, 5% CO_2_. At the end of the incubation, cells were washed three times with 3 ml ice-cold PBS and detached with 0.05% trypsin and 0.02% EDTA. Finally, all cell samples were transferred in falcon tubes and sonicated at 30% of power for 30 s in ice. The boron content of each cell sample lysates was determined by ICP-MS technique (see below). The milligrams of proteins, proportional to the number of cells, of each cell sample lysate, was evaluated by the Bradford assay (Biorad) using bovine serum albumin as a standard.

### Cell irradiation

75 flasks of MCF7 (4 flasks), ZL34 (4 flasks) and AB22 (4 flasks) were seeded at a density of 1.8 × 10^6^ cells, 1.2 × 10^6^ cells and 5.5 × 10^5^ cells, respectively. 24 h later for MCF7 and ZL34 and 72 h later for AB22, 2 flasks of each cell lines were incubated for 24 h in the presence of 72 µM, 187.5 µM and 130 µM of CA-SF, respectively. After the incubation, cells were washed with PBS and their medium was renewed. Two flasks of MCF7, ZL34 and AB22 cells incubated with CA-SF and two flasks containing non-treated control cells were irradiated for 15′ at 30 kW reactor power in the thermal column of the TRIGA Mark II reactor at the University of Pavia, Italy. At the end of the irradiation, the medium was removed, it was replaced with fresh medium and flasks were placed at 37 °C in a humidified atmosphere of 5% CO_2_.

### Proliferation assay

The proliferation assay was carried out following the same procedure reported in reference 32. The day after irradiation cells were detached with 0.05% trypsin and 0.02% EDTA and the trypan blue exclusion test of cell viability was performed. Cell viability was reported as percentage of cells observed in treated a/o irradiated samples relative to that observed in control cells.

Then, around 7 × 10^5^ MCF7, 3.5 × 10^5^ ZL34 and 1 × 10^5^ AB22 cells from each differently treated flask were seeded in 10 cm diameter culture dishes. The growing of cells was followed for at least 2 weeks and at predetermined times they were washed with PBS, detached with 0.05% trypsin and 0.02% EDTA and transferred into falcon tubes. Then, cells were sonicated for 30 s at 30% power in ice and the total cell protein concentration (that is proportional to the number of cells) from cell lysates was determined by the Bradford method, using bovine serum albumin as a standard. The Cell Growth Rate was calculated as follows:

$$\left[ {\left( {{\text{Number of cells }}\left( {t_{i} } \right) \, - {\text{Number of cells }}\left( {t_{0} } \right)} \right)/{\text{Number of cells }}\left( {t_{0} } \right)} \right] \times {1}00$$

for which Number of cells (*t*_*i*_) is the number of cells measured at the various time intervals and the number of cells (*t*_0_) is the number of cells at the starting point (t = 0) of the proliferation assay.

### CA-SF treatment on an AB22 mesothelioma tumour bearing mice and biodistribution study

Adult male Balb/c mice were maintained under specific pathogen-free conditions at the animal facility of the Department of Molecular Biotechnology and Health Sciences (Turin University, Italy). All animal experiments have been carried out in accordance with the EU Directive 2010/63/EU for animal experiments. The animal treatment protocol was approved by the Italian Ministry of Health (Authorization Number 1012/2015-PR). AB22 cells were cultured as described above and tumours were generated by the injection of 4.8 × 10^6^ AB22 cells in a final volume of 0.15 ml GFNR Matrigel (CORNING Ref. 354234): PBS (1:1 v/v) subcutaneously in the neck of the mouse. One week after the AB22-cell injection, the mice developed solid tumours of approximately 50 ± 10 mm^3^ in volume. At this time, mice (n = 6) received an intravenous injection of CA-SF (88 mg/kg corresponding to 37.5 mg/kg of ^10^B). Before the injection, CA-SF in DMSO was mixed in the presence of poly β-cyclodextrin (MW = 15,000 Da) previously dissolved in 0.9% solution of NaCl. The used CA-SF molar ratio with respect to β-cyclodextrin oligomer was 10:1. The DMSO concentration in 0.9% NaCl was kept under 5%. The mice were then sacrificed at 6 h (n = 3) and at 16 h (n = 3). The boron content of mice plasma, tumours, organs (Liver, Lung, Brain, Spleen, Kidneys) and healthy tissues (Muscle) was determined by ICP-MS technique (see below).

### Inductively coupled plasma mass spectrometry (ICP-MS)

Boron content from cell samples, mice organs, plasma and tumours were determined using inductively coupled plasma mass spectrometry (ICP-MS) (Element-2; Thermo-Finnigan, Rodano (MI), Italy).

Sample digestion was performed using a high performance Microwave Digestion System (ETHOS UP Milestone, Bergamo, Italy) after the addition of concentrated HNO_3_ (70%) to cell lysates (1:1), in a final volume of 0.4 mL. Boron content in cell samples determined by ICP-MS were normalised to the protein content of each cell sample that was correlated to the number of cells by means of a calibration curve: [(mg protein)/(number of cells)]. The amounts of boron µg /g tissue were thus calculated considering that 1 g of tissue contains 1 × 10^9^ cells.

The mice blood was collected and centrifuged at 2500 rpm for 10 min to split the plasma part over whole blood; the tumours, organs and healthy tissues were explanted. All was weighted, dissolved in HNO_3_ (70%) and digested. Both natural boron abundant and ^10^B enriched standard solution was analyzed during sample runs in order to check for variations in the systematic bias. The calibration curve was obtained using four boron absorption standard solutions (Sigma-Aldrich) in the range 0.1–0.004 μg/mL.

### BNCT treatment on AB22 mesothelioma tumour bearing mice

CA-SF treatment on mesothelioma bearing mice, were performed in the thermal column of the TRIGA Mark II reactor at Pavia University (Italy) using the same protocol reported in reference^35^. The irradiation facility was previously designed for TAOrMINA treatment developed to treat multiple liver metastases with BNCT. The chamber used to perform animal irradiation has a cross section of 40 × 20 cm^2^, a length of 1 m and it starts at about 1.3 m from the center of the reactor core. The animal irradiation position has been characterised in terms of neutron spectrum and background photon dose^43^. To carry out neutron irradiation animals are positioned at the end of this chamber where the in air thermal neutron flux is approximately 1.2 × 10^10^ n/cm^2^s, operating the reactor at its maximum power. In this way the thermal neutron flux is maximised and the irradiation time is kept as short as possible (never longer than 15 min). The first mice group (irradiated and treated group, n = 5) received three doses of CA-SF/poli β cyclodextrin (37.5 mg boron/kg dose) prepared as described before. The first injection was performed a week after tumour cells inoculation (day 0); the second injection 16 h before irradiation (day 2) and the last injection 3 days after neutron irradiation (day 6). The second mice group (irradiated group n = 5) and the third mice group (control group, n = 5) received at the same time the same volume β-cyclodextrin oligomers only in 0.9% saline solution. The fourth mice group (treated group) received three doses of CA-SF/poli β cyclodextrin (37.5 mg boron/kg dose) but mice were not irradiated. As the neutron field of the TRIGA Mark II is not collimated, the whole body of the animal is exposed to the neutron field during the irradiation. In order to reduce neutron exposure of healthy organs, a shield made of 95% ^6^Li-enriched Li_2_CO_3_ powder was used as neutron absorber. Lithium-6 is an ideal isotope to build effective neutron shields for “in vivo” experiments thanks to the absence of secondary gamma radiation after thermal neutron capture. The design of the treatment plan was carried out using the simulation code Monte Carlo N-Particles (MCNP). The validation of the simulation was performed with neutron flux measurements by the activation of Cu wires using the Westcott formalism. In the protocol used during the irradiation experiments, 5 mice were irradiated at the same time, each mouse was protected by two units of Li_2_CO_3_ neutron shield to cover the head and the abdomen regions. The units are kept separated of about 1 cm to guarantee the direct exposure of the tumours to the neutron flux.

### MRI

The T_2_-weighted MR images were acquired at 1 T with an Aspect M2-High Performance MRI System (Aspect Magnet Technologies Ltd., Netanya, Israel) consisting of a NdFeB magnet, equipped with a 35 mm solenoid Tx/Tr coil of inner diameter 35 mm. This system is equipped with fast gradient coils (gradient strength, 450 mT m − 1 at 60 A; ramp time, 250 μs at 160 V) with a field homogeneity of 0.2 − 0.5 gauss. The MR images were performed by using a T_2_-weighted protocol (TR/TE/NEX = 250:50:6; FOV = 4.0 cm). Animals were anesthetised before MRI examination by injecting tiletamine/zolazepam (20 mg/kg; Zoletil 100, Virbac, Milan, Italy) and xylazine (5 mg/ kg; Rompun, Bayer, Milan, Italy). The tumour volume (mm^3^) was calculated in the region of interest (ROI) manually drawn on the whole tumour by ITK-SNAP software. Tumour volume enhancement (%) was calculated according to the following equation: ((tumour volume (time = n) – tumour volume (time = 0)/tumour volume (time = 0)) × 100, where time = 0 indicates the day of the first CA-SF treatment and time = n indicates the days when mice were acquired by MRI.

## Results and discussion

The synthesis of the carborane functionalised sulfonamide (CA-SF) has been carried out following the procedure reported in detail in Material and Methods according to the synthetic procedure reported by Brinda et al.^14,16^ The intermediates and the final product have been fully characterised by spectroscopic methods and mass spectrometry (see Materials and Methods).

The experimental design to test the proposed methodology consisted of different steps, namely (i) assessment of CAIX expression in the considered tumour cell lines (breast and mesothelioma); (ii) in vitro assessment of breast and mesothelioma tumour cell vitality and of the cellular uptake in the presence of increasing amounts of CA-SF; (iii) BNCT experiments on tumour cells that have been loaded with CA-SF and (iv) BNCT on a mesothelioma murine model upon the i.v. administration of CA-SF.

### CAIX expression in tumour cells

**Figure 2:**
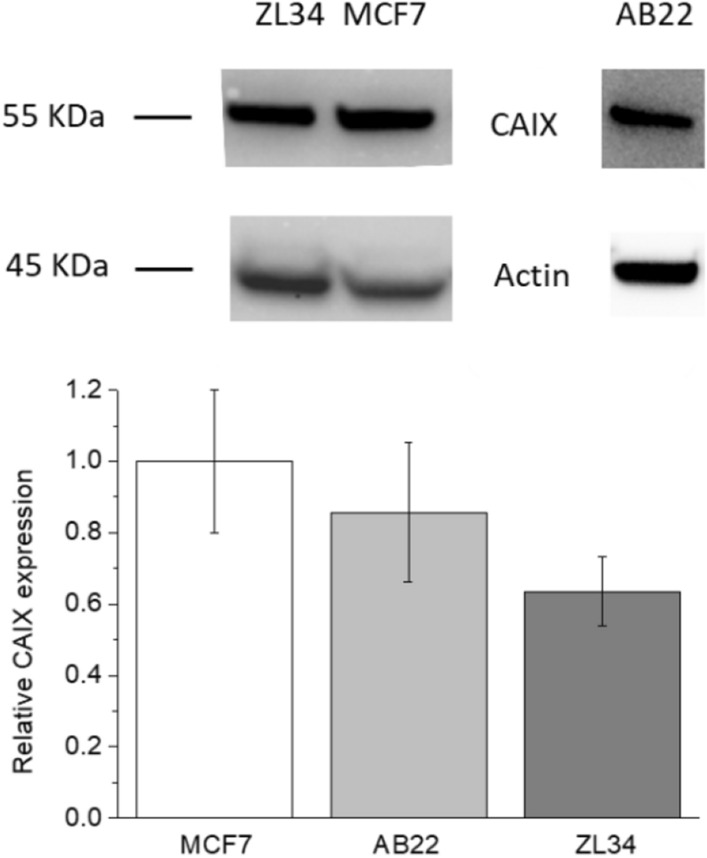
Representative Western blot experiment (upper panel): ZL34 and MCF7 were blotted separately from AB22. Analysis of expression of CAIX protein in MCF7, AB22 and ZL34 cell lines. The histograms (lower panel) represent the mean (± SE) values from three independent experiments.

The expression of CAIX was evaluated in MCF7 human breast cancer cells and in AB22 and ZL34 murine and human mesothelioma cells, respectively, by Western blot analysis (Fig. 2). The CAIX expression appeared high in both mesothelioma and breast tumours, being more intense in MCF7 and AB22 cells. The full length blots are reported in Supplementary Figure S2.

### Cell vitality assay

**Figure 3:**
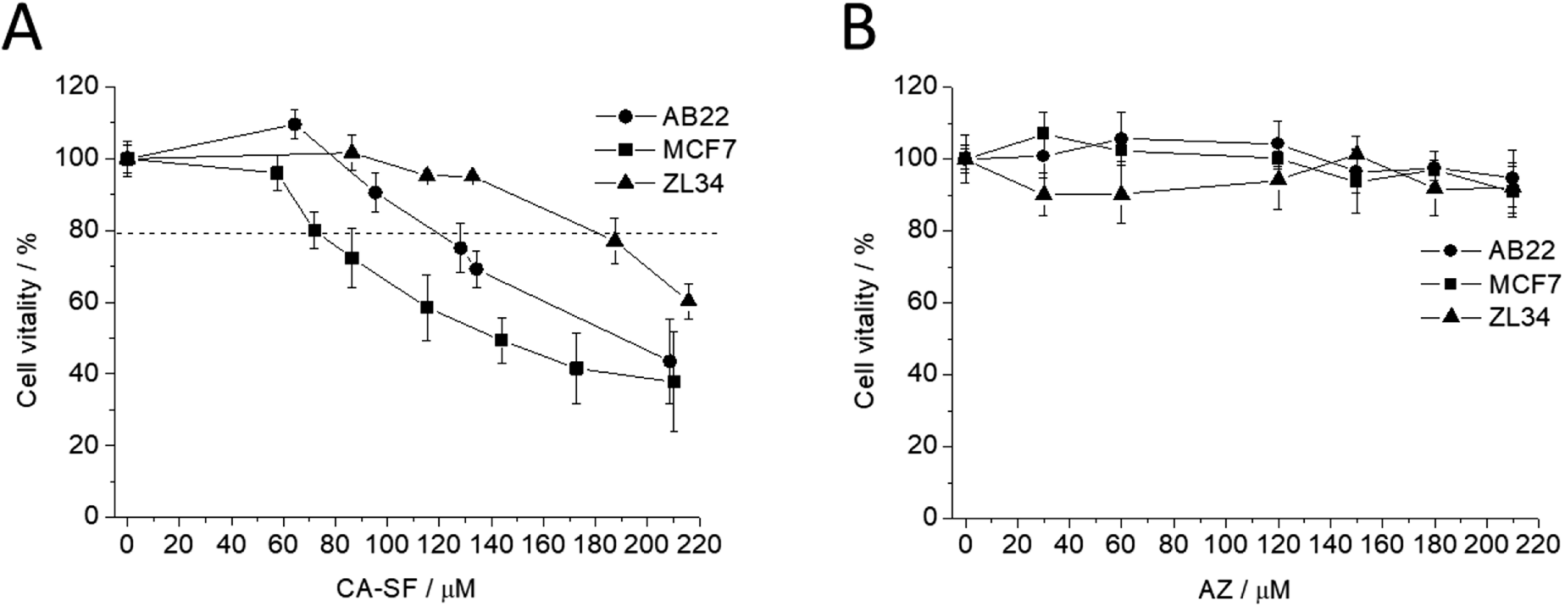
Cell vitality assay (MTT) performed on MCF7, ZL34 and AB22 cells incubated for 24 h at 37 °C in normoxia with increasing concentration of CAIX inhibitors (CA-SF) and acetazolamide (AZ). Error bars indicate ± SD.

The in vitro assessment of the enzyme-inhibitory capacity of CA-SF against CAIX was performed by evaluating cell vitality by means of the MTT assay. The observed activity of CA-SF was compared with acetazolamide (AZ), a clinically approved CA inhibitor^9,44^. The experiments were carried out by adding increasing amounts of CA-SF or AZ to the incubation medium containing the cells (MCF7, ZL34 and AB22, respectively) for 24 h in normoxic condition (at 37 °C and 5% CO_2_ in a cell incubator). Figure 3A reports that the percentage of viable cells steadily decreases upon increasing the concentration of CA-SF thus demonstrating its toxic effect on cells overexpressing CAIX enzyme. MCF7 and AB22 cells appear the better responding cells as expected on the basis of their higher CAIX expression. Conversely, AZ exhibited a negligible enzyme-inhibitory capacity for the tested cell lines when incubated in the same concentration range (Fig. 3B). This finding may be accounted for the relatively high IC50 (> 800 μM) reported in the literature for MCF7 cells when incubated for a longer time (48 h)^44^. This result outlines the important role of carborane to increase the affinity to the target enzyme thus improving the inhibitor capability of the sulfonamide group.

### Cell uptake experiments

**Figure 4:**
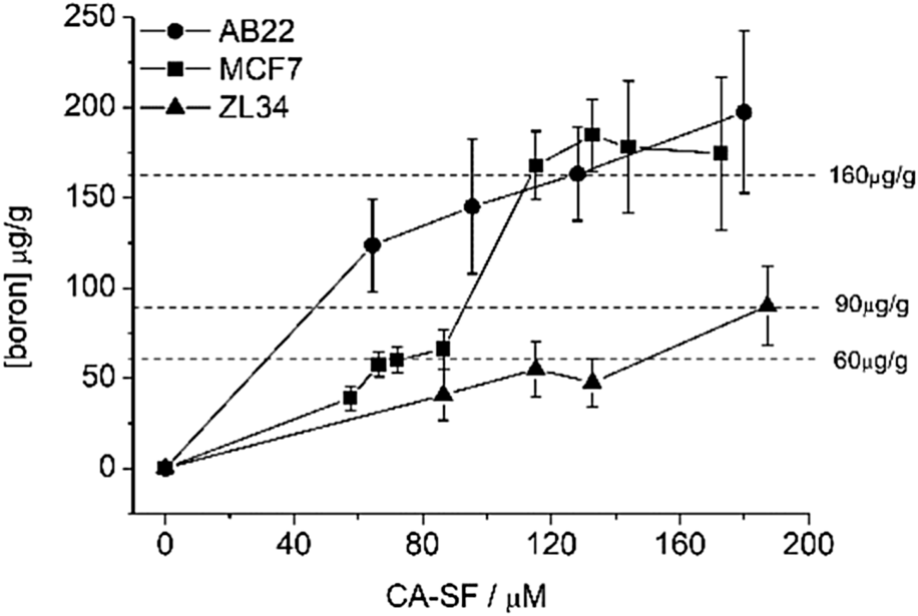
Upload of boron in MCF7, ZL34 and AB22 cells incubated in the presence of increasing concentration of CA-SF for 24 h at 37 °C. Error bars indicate the SD.

In order to assess whether the amount of boron taken-up by target cells was enough to allow the set-up of an efficient BNCT procedure, MCF7, ZL34 and AB22 cells incubated with increasing concentration of CA-SF, were analyzed for their boron content by ICP-MS technique. Figure 4 shows that CA-SF concentrations of 72 µM, 187.5 µM and 130 µM for MCF7, ZL34 and AB22, respectively, in the culture medium were sufficient to load 60, 90 or 160 µg/g of boron into the target cells after 24 h incubation at 37 °C, 5% CO_2_. These quantities appear well sufficient to perform BNCT. Under these experimental conditions, the cell vitality measured by MTT was about 80% for all the considered cell lines.

To assess whether the combination of BNCT and CAIX enzyme-inhibitory capacity may result in an improvement of the treatment outcome with respect to CAIX enzyme-inhibitory capacity given as monotherapy, MCF7, ZL34 and AB22, previously treated with CA-SF, were irradiated with thermal neutrons at the TRIGA Mark II reactor at the University of Pavia.

### BNCT treatment of MCF7, ZL34 and AB22 cells in vitro

**Figure 5:**
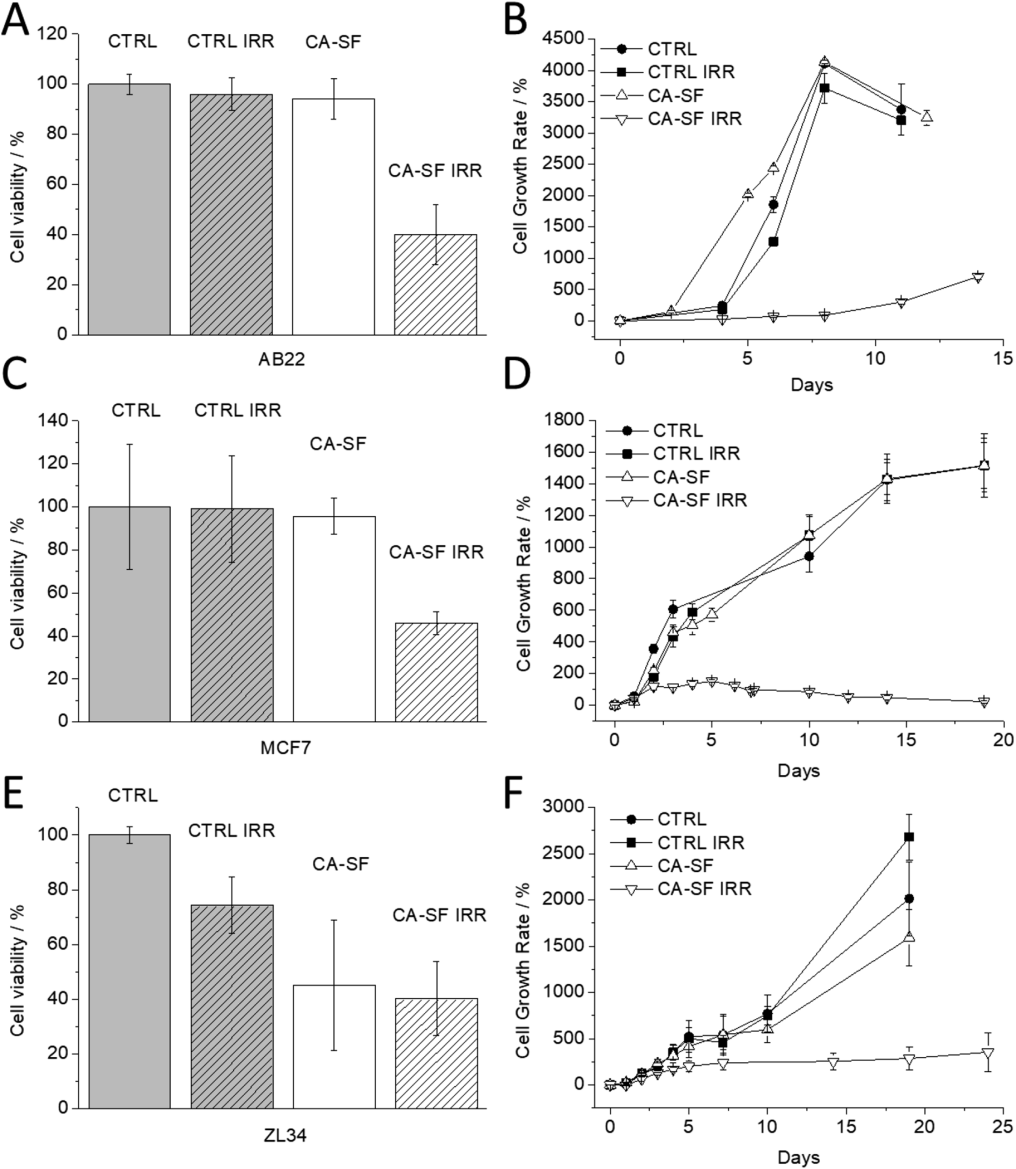
(**A**, **C**, **E**) Number of viable cells 24 h after neutron irradiation. (**B**, **D**, **F**) Effect of the irradiation after CA-SF (containing ten boron atoms) treatment on breast (MCF7) and mesothelioma cells lines ZL34 and AB22 (proliferation rate of cells surviving to irradiation). Error bars indicate the SD.

The experimental plan consisted of comparing the vitalities of irradiated and not irradiated cells. The first two groups were represented by untreated cells (CTRL IRR) and CA-SF treated cells (CA-SF IRR) both irradiated with thermal neutrons. The results on these cells were compared with the corresponding not irradiated ones, i.e. those denoted as (CTRL) and (CA-SF), respectively. Boron treated cells were incubated for 24 h with a CA-SF concentration of 72 µM, 187.5 µM and 130 µM for MCF7, ZL34 and AB22, respectively, in order to deal with a similar cytotoxicity values for the three considered cell lines. The neutron-irradiated groups (CTRL IRR + CA-SF IRR) were exposed for 15′ minutes to the radiation field of the thermal column of the TRIGA Mark II reactor (reactor power 30 kW). Figure 5 shows the percentage of cells that survived to neutron irradiation (CA-SF loaded and unloaded) with respect to the not irradiated control cells (CTRL and CA-SF groups). The histograms of AB22 (Fig. 5A) and MCF7 (Fig. 5C) show a marked difference between CA-SF treated cells and CA-SF treated and irradiated cells. From these results, it is evident that the viability of cells treated with boron containing CA-SF was significantly reduced upon the irradiation treatment. Only for ZL34 cells (Fig. 5E), the number of viable cells survived to CA-SF treatment, were comparable to those ones survived to both CA-SF treatment and irradiation. Moreover, the proliferation rate of cells which survived to irradiation (Fig. 5B,D,F) revealed an interesting behavior. Only survived cells that were treated with the CA-SF before neutron irradiation showed a marked inhibition of proliferation. This result supports the view that CAIX inhibitors may have a relevant role to hamper the proliferation of tumour cells undergone to the BNCT treatment.

### In vivo tumour growth assessment upon application of BNCT to CA-SF administered mice

The in vivo study was carried out on a syngeneic mouse model obtained by subcutaneous implantation, at the bottom of the neck, of AB22 cells. Among different cell lines, mesothelioma was the selected one because, to date, there is no curative treatment for this disease and the available therapies can guarantee an expected survival time of less than one year^45–47^. Moreover, the in vitro experiments demonstrated a high boron accumulation (Fig. 4) compared to ZL34 and MCF7 cells. In order to inject the CAIX inhibitor in a bolus of limited volume, the low solubility of CA-SF in aqueous media was improved by forming an inclusion complex with a β-cyclodextrin oligomer (MW = 15,000)^48^. Cyclodextrins are cyclic oligosaccharides with a truncated cone-shaped structure consisting of a hydrophobic inner cavity and a hydrophilic outer surface. The cyclodextrins are widely used for increasing the aqueous solubility of hydrophobic compounds as well as to promote the slow release of the encapsulated compounds in the region where they distribute^49^. The higher molecular weight of the β-cyclodextrin-oligomer is expected to favor the selective extravasation in tumour tissue due to EPR effect (Enhanced Permeation and Retention) and a slower clearance from the pathological site. On can surmise that the use of a polymeric-cyclodextrin yields also a prolonged release of the encapsulated CA-SF molecules, although this aspect was not investigated in this work^50^.

The tumour bearing mice were prepared by injecting ca. 5 million AB22 cells subcutaneously in Balb/c mice (n = 6). After one week, the AB22 tumours reached a volume of approximately 50 ± 10 mm^3^. At this time, a dose of CA-SF (37.5 mg/kg of boron) under the form of a supramolecular adduct with β-cyclodextrin-oligomer (CA-SF/Poly-CD molar ratio of 1:10.) was i.v. injected. The boron concentration at the target tumour tissue was measured ex vivo by ICP-MS, 6 and 16 h after the injection, respectively.

**Figure 6:**
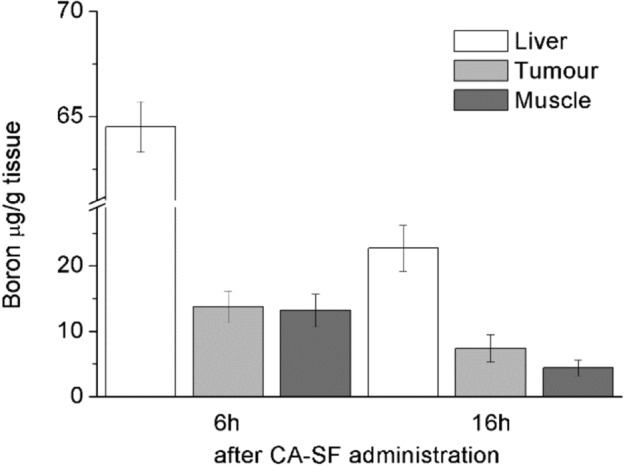
Boron biodistribution at 6 and 16 h in AB22 tumour bearing mice after the administration of CA-SF. Boron concentration was determined by ICP-MS. Error bars indicate the SD.

A high boron concentration in tumour needs to be combined with a high tumour/muscle boron ratio that is fundamental to reduce healthy tissue damage during BNCT. As reported in Fig. 6, the highest tumour to healthy tissue boron ratio (1.7:1, Table 1) was obtained 16 h after the CA-SF administration. Moreover, the amount of boron in all the other organs and plasma was dramatically reduced after 16 h compared to 6 h (Table 1). On this basis, BNCT was performed 16 h after CA-SF injection.

**Table 1:** Biodistribution of boron concentration in AB22 mesothelioma tumours bearing mice after CA-SF administration. Boron concentration was determined by ICP-MS. Error bars indicate the SD.

Times post i.v. (h)	Brain [boron] μg/g ± SD	Kidney [boron] μg/g ± SD	Liver [boron] μg/g ± SD	Lungs [boron] μg/g ± SD	Plasma [boron] μg/g ± SD	Spleen [boron] μg/g ± SD	Tumour [boron] μg/g ± SD	Muscle [boron] μg/g ± SD	Ratio of boron in tumour/muscle
6	30.7 ± 11.2	48.2 ± 15.3	64.5 ± 1.2	37.2 ± 11.2	20.4 ± 5.4	18.5 ± 5.8	13.8 ± 2.4	13.2 ± 2.5	1:1
16	7.3 ± 1.1	13.7 ± 1.9	22.7 ± 3.5	12.1 ± 2.2	11.1 ± 1.3	6.2 ± 1.5	7.4 ± 2.1	4.4 ± 1.2	1.7:1

To evaluate the effectiveness of CA-SF on AB22 tumour growth and to exploit the advantage of the combined CA-SF + BNCT treatment, two groups of AB22 tumour bearing mice were considered, namely the group treated only with CA-SF (CA-SF group, n = 5) and the one treated with CA-SF and irradiated with thermal neutrons (CA-SF-IRR group, n = 5). The treatment with CA-SF was performed by multiple doses of the CAIX inhibitor. The two groups of mice (CA-SF and CA-SF + IRR groups) received three doses of CA-SF (37.5 mg/kg of boron), namely at day 0, when tumours reached 50 ± 10 mm^3^, at day 2 and at day 6. CA-SF-IRR group was irradiated with neutrons at day 3 (16 h after the second CA-SF administration). These groups were compared to the control group that was treated with β-cyclodextrin oligomers only in 0.9% saline solution and not irradiated (CTRL group, n = 5) and to the irradiated group (IRR group, n = 5) that received neutron irradiation at day 3 after the administration β-cyclodextrin oligomers in 0.9% saline solution only. Both IRR and IRR + CA-SF groups were irradiated for 15′ in the thermal column of the TRIGA Mark II reactor (reactor power 250 kW).

The weight of IRR, CA-SF and CA-SF + IRR groups was monitored till the end of the experiment (Supplementary Figure S1).

**Figure 7:**
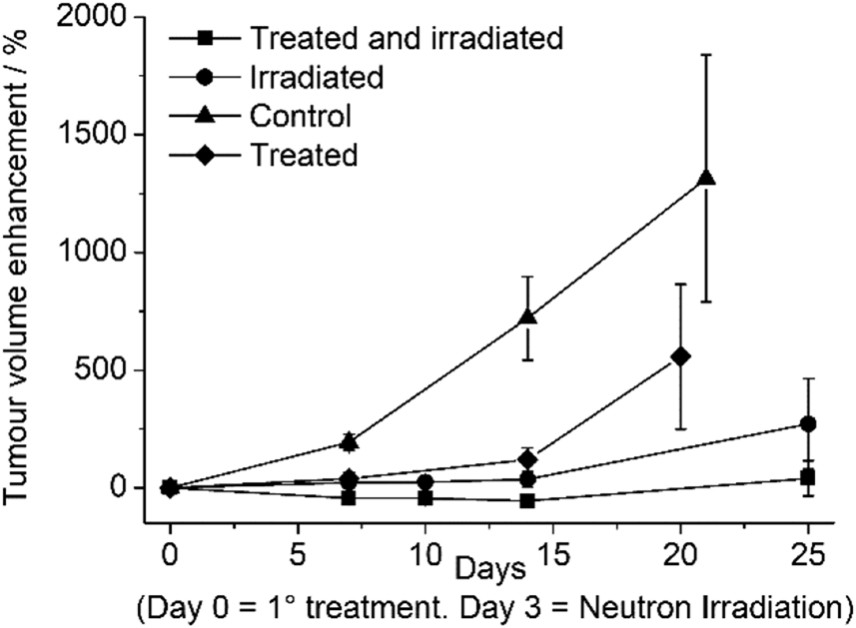
Tumour-growth evaluation performed after CA-SF treatment. The graph shows the tumour volume increase measured by MRI on control mice, CA-SF treated mice, irradiated mice and irradiated and treated mice. Error bars indicate the SE.

**Figure 8:**
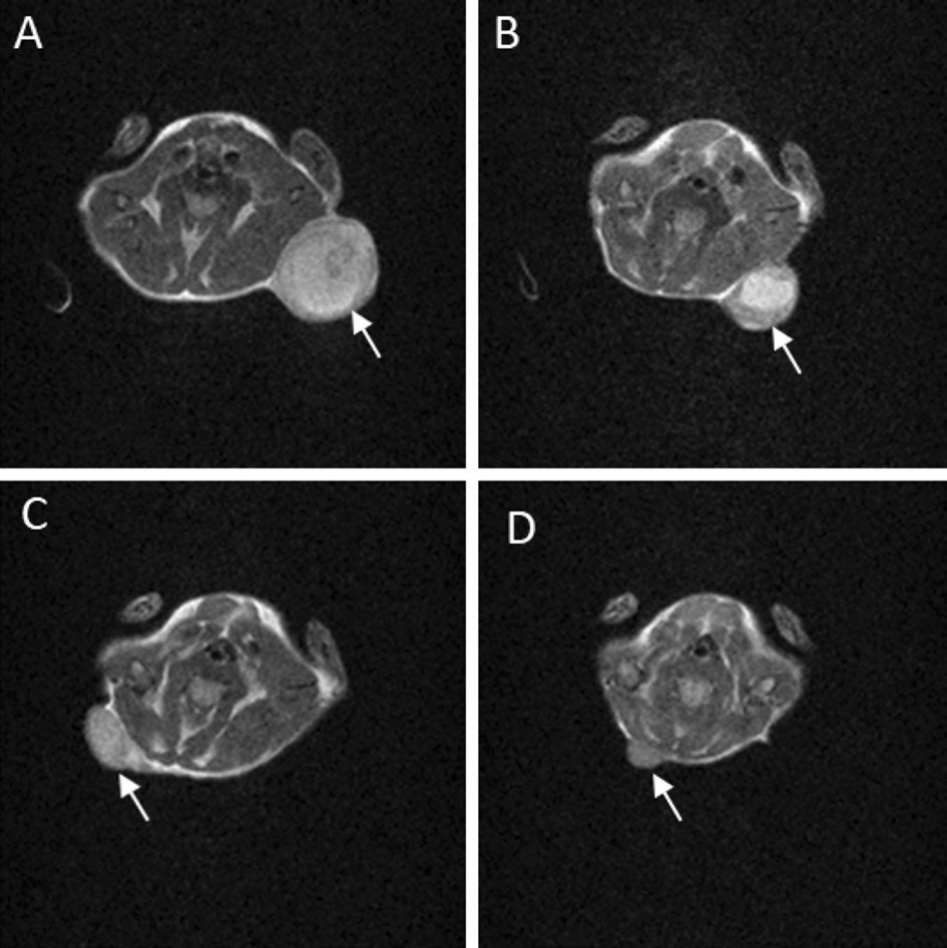
T_2_-weighted images (1 T) acquired at day 14 on untreated (**A**) treated with CA-SF (**B**), irradiated (**C**) or treated with CA-SF and irradiated (**D**) AB22 tumour bearing mice. The arrows indicate the tumour region.

Tumour volume was measured for the following 25 days by analyzing T_2_ weighted Magnetic Resonance Imaging (MRI) images acquired at 1 T. Figures 7 and 8 show that the CA-SF administration significantly inhibits mice tumour growth (treated mice) with respect to control mice treated with the same volume of the vehicle alone demonstrating the effectiveness of CA-SF as CAIX inhibitor. Treated and irradiated mice curve show that the synergic CA-SF + BNCT treatment resulted in a dramatic reduction of the mass of tumours till 25 days from the first CA-SF administration. BNCT enhanced the effectiveness of CA-SF therapy alone succeeding in maintaining negligible the tumour growth till the end of the study. Interestingly, the irradiated mice (w/o CA-SF) showed a significant decrease of the tumour volumes with respect to control and treated mice groups. This behavior could be related to the activation of mice immune response after the neutron irradiation. After 15 days, only in treated and irradiated mice the tumours growth still remained negligible while in all the other groups the tumours started to re-growth.

## Conclusions

In this study, it has been shown that CA-SF (carborane containing carbonic anhydrase inhibitor) is able to hamper the growth of mesothelioma and breast cancer cells, as expected on the basis of its binding to CAIX overexpressed in these cells. Moreover, its cellular uptake has a synergic toxic effect upon the application of BNCT. Most important, the “in vivo” results showed a very limited mesothelioma tumour re-growth in the case of CA-SF treated and irradiated mice. This finding supports the view that combination of BNCT with targeted cellular therapies may pave the way for new approaches to cure cancer patients. It is of great importance to develop new boronated derivatives to be used in BNCT. This appears particularly relevant nowadays when we are entering in the “new era” of BCNT. In fact one main drawback that prevented the diffusion of BNCT as a routine therapeutic protocol was due to the access to reactors as they were the only neutron sources able to yield neutron beams suited for therapy. Today the technology is ready to produce neutron beams from high-current proton accelerators coupled with Li or Be targets, and the possibility of installing these machines in hospitals makes BNCT a more accessible option. Indeed, two accelerator-based BNCT clinical facilities are already operating in Japan^51^, and other projects to install such facilities are underway in Japan, Finland, Argentina, China and Italy.

## Supplementary information

Supplementary Information
