# Supplementary material for: In vitro and in vivo BNCT investigations using a carborane containing sulfonamide targeting CAIX epitopes on malignant pleural mesothelioma and breast cancer cells
Source: Sci Rep. 2020 Nov 6;10:19274. doi: 10.1038/s41598-020-76370-1 (PMC7648750; doi:10.1038/s41598-020-76370-1)
Supplement: Supplementary file 1 — Supplementary Information [file 41598_2020_76370_MOESM1_ESM.docx]

**Supporting Information**

***In vitro* and *in vivo* BNCT Investigations using a Carborane containing Sulfonamide Targeting CAIX epitopes on Malignant Pleural Mesothelioma and Breast Cancer Cells**

Diego Alberti,^a^ Alessia Michelotti^b^, Alberto Lanfranco^b^, Nicoletta Protti,^c,d^ Saverio Altieri^c,d^, Annamaria Deagostino.^b^* Simonetta Geninatti Crich^a^*

**

**After BNCT treatment, the weight of irradiated, treated, and treated and irradiated mice was monitored till the end of the experiment. The graph shows that only in the case of treated and irradiated mice, the animals lost only 10% of their initial weight before irradiation, marginally affecting the weight increment in the first 10-15 days followed by a slow but constant weight recover in the last 10 days. In the case of irradiated group, the mice weight continued to increase, demonstrating that 15’ irradiation didn’t affect their weight. Treated mice weight curve shows only a negligible weight descrease of 5% measured at the end of the study.

**Figure S1**: Mice weight vs. time after irradiation (Days). The irradiation time was of 15’ at a reactor power of 250 kW. Error bars indicate the SE.


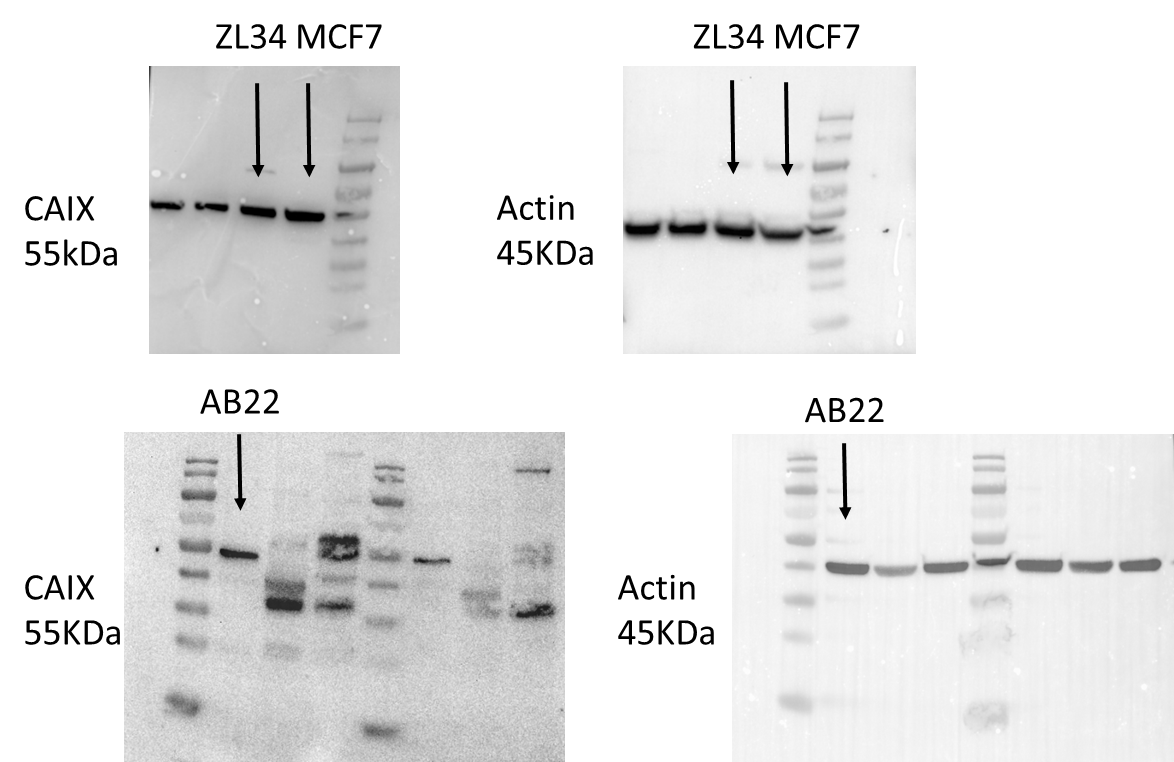


**Figure S2**: Representative Western blot experiment: full-length blots of Figure 2.

**Spectral characterization of ^10^B-emriched-1-sulfonamidomethyl–o-carborane (CA-SF).**

**^1^H, ^13^C and DEPT spectra**

**IR**

**
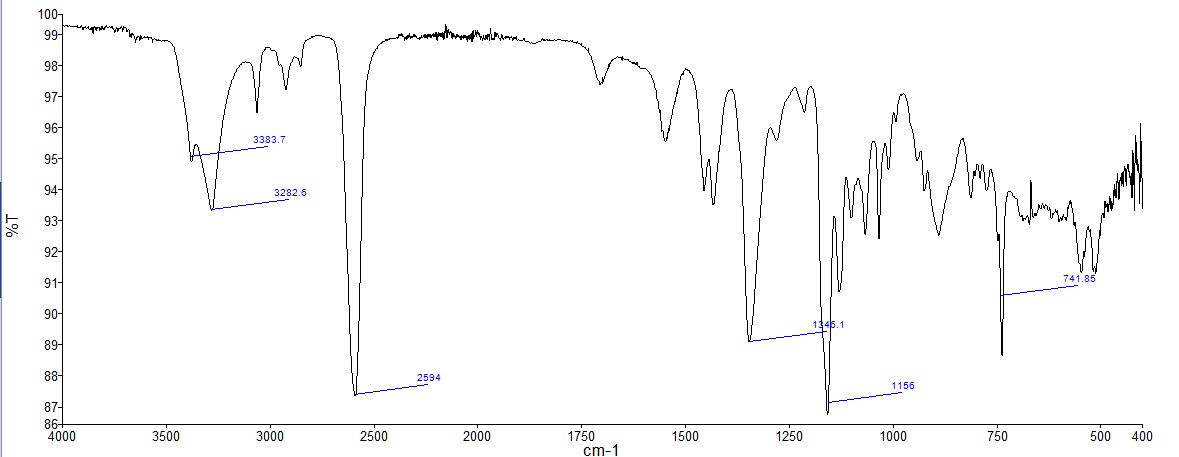
**

**Exact mass**

**C_3_H_16_B_10_N_2_O_2_S**
